# Contrasting free-living and particle-associated aerobic anoxygenic phototrophic bacterial communities across space and time in the Alboran Sea

**DOI:** 10.1093/ismeco/ycag120

**Published:** 2026-04-29

**Authors:** Jorge J Santos-Bruña, Carlota R Gazulla, Ana M Cabello, Candela García-Gómez, Soluna Salles, M Teresa Camarena-Gómez, Miriam Domínguez-Rodríguez, Antonio Sánchez, Nerea Valcárcel-Pérez, Francisco Gómez-Jakobsen, Seyed Mohammad Sadeghi-Nassaj, Isabel Reche, Barbara Marie, Eva Ortega-Retuerta, Lidia Yebra, Jesús M Mercado, Isabel Ferrera

**Affiliations:** Centro Oceanográfico de Málaga, Instituto Español de Oceanografía, IEO-CSIC, 29002 Málaga, Spain; Centro Oceanográfico de Málaga, Instituto Español de Oceanografía, IEO-CSIC, 29002 Málaga, Spain; Centro Oceanográfico de Málaga, Instituto Español de Oceanografía, IEO-CSIC, 29002 Málaga, Spain; Centro Oceanográfico de Málaga, Instituto Español de Oceanografía, IEO-CSIC, 29002 Málaga, Spain; Centro Oceanográfico de Málaga, Instituto Español de Oceanografía, IEO-CSIC, 29002 Málaga, Spain; Centro Oceanográfico de Málaga, Instituto Español de Oceanografía, IEO-CSIC, 29002 Málaga, Spain; Centro Oceanográfico de Málaga, Instituto Español de Oceanografía, IEO-CSIC, 29002 Málaga, Spain; Centro Oceanográfico de Málaga, Instituto Español de Oceanografía, IEO-CSIC, 29002 Málaga, Spain; Centro Oceanográfico de Málaga, Instituto Español de Oceanografía, IEO-CSIC, 29002 Málaga, Spain; Centro Oceanográfico de Málaga, Instituto Español de Oceanografía, IEO-CSIC, 29002 Málaga, Spain; Departamento de Ecología and Instituto del Agua, Facultad de Ciencias, Universidad de Granada, 18071 Granada, Spain; Departamento de Ecología and Instituto del Agua, Facultad de Ciencias, Universidad de Granada, 18071 Granada, Spain; Laboratoire d'Ecogéochimie des Environnements Benthiques (LECOB), Observatoire Océanologique de Banyuls, CNRS, Sorbonne Université, 66650 Banyuls-sur-Mer, France; Laboratoire d'Ecogéochimie des Environnements Benthiques (LECOB), Observatoire Océanologique de Banyuls, CNRS, Sorbonne Université, 66650 Banyuls-sur-Mer, France; Centro Oceanográfico de Málaga, Instituto Español de Oceanografía, IEO-CSIC, 29002 Málaga, Spain; Centro Oceanográfico de Málaga, Instituto Español de Oceanografía, IEO-CSIC, 29002 Málaga, Spain; Centro Oceanográfico de Málaga, Instituto Español de Oceanografía, IEO-CSIC, 29002 Málaga, Spain

**Keywords:** AAP bacteria, Alboran Sea, *pufM* gene, free-living bacteria, particle-associated bacteria

## Abstract

Aerobic anoxygenic phototrophic (AAP) bacteria play an important role in ocean biogeochemistry by degrading organic matter and capturing light energy through bacteriochlorophyll *a*. However, how their genetic and ecological traits vary with lifestyle remains underexplored. We investigated free-living (FL) and particle-associated (PA) AAP communities and their responses to environmental variability in the northern Alboran Sea. Surface seawater was collected during seven cruises conducted in spring, autumn, and winter, each covering four coast-to-offshore transects. AAP abundance was quantified by infrared epifluorescence microscopy, and community diversity was assessed using *pufM* gene amplicon sequence variants (ASVs). AAP abundance was significantly higher in spring than in winter, although no consistent spatial pattern was observed. PA communities exhibited greater diversity than FL communities. Around 65% of ASVs were unique to the PA fraction, although most of these were rare. Distinct AAP phylogroups displayed clear niche preferences: PA-associated groups were more common in coastal waters, whereas FL groups predominated offshore. Both communities showed pronounced seasonality, but spatial structuring along the west-to-east gradient was more apparent in FL than in PA communities. Environmental drivers also differed between lifestyles, with salinity primarily shaping FL communities and chromophoric dissolved organic matter influencing PA communities. Our results indicate that lifestyle (FL vs. PA) is a major determinant of AAP bacterial diversity, spatiotemporal patterns, and environmental responses.

## Introduction

Aerobic anoxygenic phototrophic (AAP) bacteria are facultative photoheterotrophs that grow mainly on dissolved organic matter (DOM), but also use bacteriochlorophyll *a* to harvest light energy and supplement their metabolism [1, 2]. These bacteria are widespread in the upper ocean, typically accounting for <1%–8% of bacterioplankton in euphotic open waters [3–5], but their relative abundance can increase in eutrophic environments [6, 7]. Due to their rapid growth and susceptibility to grazing [8], AAP bacteria likely play an important role in ocean biogeochemistry, particularly in the carbon cycle [9].

The diversity of AAP bacteria has traditionally been investigated through polymerase chain reaction (PCR) amplification of the *pufM* gene, which encodes a subunit of the photosynthetic reaction center. Based on this gene, marine AAP bacteria, mainly Alphaproteobacteria and Gammaproteobacteria, display variable patterns of diversity and distribution across environments, oceanic regions, and seasons [10–12]. In the Mediterranean Sea, diversity shows an inverse relationship with the dominance of phylogroup K (Pseudomonadales), which consistently prevails in spring [10, 11, 13]. Additionally, a positive correlation between AAP abundance and chlorophyll *a* concentration has been reported in the Atlantic and Pacific Oceans, as well as in the Mediterranean Sea [3, 5, 14]. Coast-to-offshore transects confirm this trend, showing a decline in AAP abundance from nearshore to offshore waters [14, 15]. The higher AAP abundance observed in coastal areas may reflect their association with particles of different nature, including cell debris and terrestrial inputs [16]. AAP bacteria can occur in both free-living (FL) or particle-associated (PA) forms, with the PA fraction increasing from the open ocean toward coastal zones [17]. Only a few studies have examined how marine AAP communities relate to lifestyle. In the Chesapeake Bay, no significant differences were found between FL and PA fractions [16]. More recently, greater diversity differences were observed among photosynthetically active AAP communities than among potentially photosynthetic ones in the Adriatic Sea [18]. Nonetheless, it remains unclear whether marine AAP community composition varies with lifestyle in other marine regions, or how such variation relates to environmental conditions.

Recent findings have shown that the primers commonly used for *pufM* gene amplicon sequencing tend to overrepresent certain Gammaproteobacteria and Alphaproteobacteria clades while underestimating other abundant groups [19, 20]. Metagenomic analyses have further revealed that several uncultured phylogroups, specifically A, B, C, and D, were previously underrepresented due to primer bias [11, 20–22]. A recent comparative assessment of *pufM*-targeted primer sets identified more effective alternatives and demonstrated that phylogroups A and B can be particularly abundant in winter in the Mediterranean Sea [20].

The goal of this study is to assess the diversity and distribution of AAP communities in the northern Alboran Sea (SW Mediterranean) across different environmental conditions and lifestyles, using the most effective primers currently available. Specifically, we aim to (i) determine whether FL and PA community compositions vary along coast-to-offshore gradients throughout the seasonal cycle and (ii) identify the environmental factors that drive these patterns. To address these questions, we collected surface seawater samples during seven oceanographic cruises along the northern Alboran coast, each comprising four coast-to-offshore transects. We quantified AAP abundance using infrared epifluorescence microscopy and analyzed community diversity via *pufM* gene amplicon sequence variants (ASVs). Our results demonstrate that lifestyle (FL vs. PA) is a key determinant of AAP bacterial composition, spatiotemporal distribution, and ecological dynamics.

## Material and methods

### Sample collection and processing

**Figure 1:**
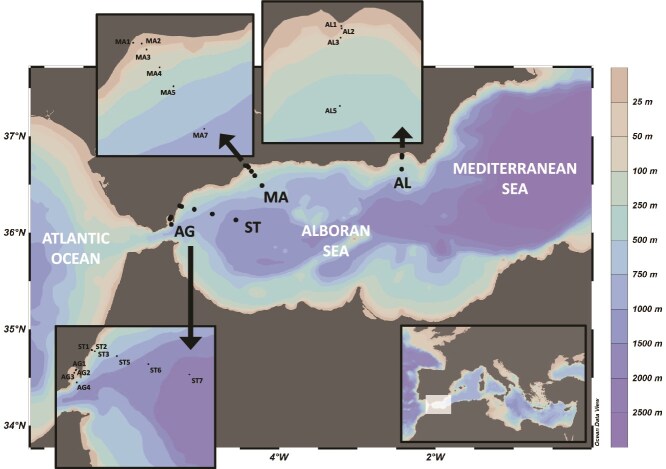
Bathymetric map of the Alboran Sea showing the four coast-to-offshore transects (AG: Algeciras, ST: Sotogrande, MA: Málaga, and AL: Almería) and the sampling stations (black dots with labels) visited during each cruise. Map created using Ocean Data View (https://odv.awi.de).

Samples were collected during seven cruises in the northern Alboran Sea: 14–18 January (ES0120) and 5–11 June (ES0620) 2020; 9–14 January (ES0121) and 15–19 May (ES0521) 2021; and 30 January–2 February (ES0122), 21–26 March (ES0322), and 24–26 November (ES1122) 2022. According to Northern Hemisphere astronomical seasons, ES0120, ES0121, ES0122, and ES0322 correspond to winter; ES0620, and ES0521 to spring; and ES1122 to autumn. Each cruise included four coast-to-offshore transects, from west to east: Algeciras (AG), Sotogrande (ST), Málaga (MA), and Almería (AL) (Fig. 1). At each station, surface seawater was collected at 3-m depth using Niskin bottles and processed onboard for the analysis of biotic and abiotic variables. Concurrently, a CTD SBE-25 recorded temperature, conductivity, and dissolved oxygen of the surface seawater at sampling stations. For organic matter analyses, samples were filtered onto pre-combusted glass fiber filters (GF/F; Whatman) using glass vacuum flasks, stored in amber glass bottles, and frozen at −20°C. For nutrient determinations, three replicates per Niskin bottle were frozen at −20°C. Concentrations of nitrate (NO_3_^−^), nitrite (NO_2_^−^), ammonium (NH_4_^+^), phosphate (PO_4_^3−^), and silicate (SiOH) were determined following Ramírez *et al*. [23] using a continuous segmented flow analyzer (QuAAtro39; SEAL Analytical). Chlorophyll *a* (Chl *a*) samples were collected by filtering 1–3 L of seawater through Whatman GF/F (total fraction) and 20-μm polycarbonate (>20 μm fraction) filters, then frozen at −20°C. Chl *a* concentration was determined by spectrophotometry after overnight extraction in 90% acetone at 4–5°C and darkness [24]. Abundances of picocyanobacteria (*Synechococcus* and *Prochlorococcus*), heterotrophic high- and low-nucleic acid (HNA and LNA) bacteria, and photosynthetic picoeukaryotes (PPEs) were quantified by flow cytometry following Gasol and Morán [25].

### Biomass collection

Biomass for DNA extraction was concentrated by pre-filtering ~2 L of seawater through a 200-μm nylon mesh to remove large plankton, followed by sequential filtration onto 3-μm and 0.2-μm polycarbonate filters (47 mm; DHI, Denmark). This procedure separated bacterioplankton into particle-associated (PA; 3–200 μm) and free-living (FL; 0.2–3 μm) fractions. To prevent clogging and minimize the retention of FL bacteria to the 3-μm filter, filters were replaced when flow rate decreased. Filters were stored at −80°C until further processing.

### A‌AP cell counts

For AAP cell counts, samples were pre-filtered through a 200-μm mesh, fixed with 3.7% formaldehyde (final concentration), and filtered onto 0.2-μm polycarbonate filters (25 mm Nuclepore, Whatman). AAP cells were enumerated in a total of 21 samples from three cruises (ES0121, ES0521, and ES0122) using infrared epifluorescence microscopy following [5, 26, 27]. Details can be found in the Supplementary Materials.

### Dissolved organic matter

Chromophoric dissolved organic matter (CDOM) was analyzed spectrophotometrically using the absorption spectra (200–750 nm) of filtered samples measured with a Perkin Elmer UV/VIS spectrophotometer. CDOM was characterized from the absorbance ratios E2:E3 (250/365 nm) and E4:E6 (465/665 nm) [28, 29], absorbance coefficients *a*_254_ and *a*_325_ [30, 31], and spectral slopes of absorbance coefficients between 275–295 nm (*S*_275–295_) and 350–400 nm (*S*_350–400_) along with their ratio (SR) [32]. The concentration of dissolved organic carbon (DOC) was analyzed from filtered samples by high-temperature catalytic oxidation [33] with a Shimadzu TOC-L-CSH analyzer, after sample acidification (pH < 2) with 85% H_3_PO_4_.

### DNA extraction, sequencing, and amplicon processing

DNA was extracted from the 3-μm and the 0.2-μm filters using the DNeasy PowerSoil Pro Kit (Qiagen), following the manufacturer’s protocol. DNA quality was checked via spectrophotometry (BioDrop Touch Duo; Biochrom), and its concentration was measured by fluorometry (Qubit; Life Technologies). DNA samples were stored at −80°C and an aliquot was sent out for sequencing. Partial amplification of the *pufM* gene (~145 bp fragment) was performed using primers pufM_uniF (5′-GGNAAYYTNTWYTAYAAYCCNTTYCA-3′) and pufM_UniR (5′-YCCATNGTCCANCKCCARAA-3′) [19]. PCR conditions were as follows: initial denaturation at 95°C for 5 min; 35 cycles of 95°C (30s), 48°C (45 s), and 72°C (45 s); and a final elongation at 72°C for 7 min. DNA sequencing was performed on an Illumina NovaSeq PE250 by AllGenetics & Biology SL (www.allgenetics.eu). A total of 26 PA samples failed to amplify (Table S1), yielding high-quality sequences from 195 samples (123 FL and 72 PA). After sequencing, primers were removed with Cutadapt v1.16, and ASVs were inferred with DADA2 v1.10 [34] using maxEE = c(2,2) and trunclen = c(145,145). DADA2 was also used to remove chimeras and spurious sequences. Moreover, AliView v1.30 [35] was used to screen for off-target amplicons performing an alignment of the ASVs from DADA2. After this quality control step, singletons or very rare ASVs were retained in the dataset. After processing, the dataset included 7020 ASVs and 2 731 290 reads (mean = 14 007 ± 3624 SD). ASV taxonomic assignment was inferred using a phylogenetic tree of *pufM* gene sequences. For this, we used the reference tree from Gazulla *et al*. [12] and incorporated *pufM* sequences from the database in Villena-Alemany *et al*. [36] as well as *pufM* sequences from the single amplified genomes generated in Pachiadaki *et al*. [37]. *Chloroflexus auranticus* was used as outgroup. Then, we performed the taxonomic assignation applying the Evolutionary Placement Algorithm v0.3.5 [38] with Gappa v0.8.4 [39]. ASVs were classified according to the phylogroups defined by Yutin *et al*. [21], supplemented with Genome Taxonomy Database (GTDB; https://gtdb.ecogenomic.org/) assignments when available. For example, phylogroups I and K corresponded to members of the Burkholderiales (including the genus *Limnohabitans*) and the Pseudomonadales (Gammaproteobacteria, including the genus *Luminiphilus* within the family Haliaceae), respectively. In contrast, phylogroup G was associated with the order Rhodobacterales (Alphaproteobacteria). Within phylogroup G, two distinct clusters were identified in the phylogenetic tree and were designated as Rhodobacterales G1 and G2. To refine taxonomic assignments, certain ASVs were aligned against the GenBank nonredundant database using BLAST (https://blast.ncbi.nlm.nih.gov/Blast.cgi). Genera were assigned based on the highest alignment accuracy, provided the taxonomy matched the previously assigned phylogroup. Overall, ASVs were classified into 14 phylogroups, including an “Others” group comprising ASVs assigned to minor Alpha- and Gammaproteobacteria clades, and an “Unclassified” group for sequences assignable only at the domain level. A total of 93 ASVs, representing 0.02% of total reads, identified as Chloroflexota (green non-sulfur bacteria) or Chromatiales (purple sulfur bacteria) were excluded, resulting in a final ASV table of 6927 ASVs and 2 729 901 reads (mean = 13 999 ± 3617 SD).

### Statistical analysis

All analyses were performed in R (v4.3.3). For alpha and beta diversity, the ASV table was rarefied down to 3020 reads per sample using the “phyloseq” package [40], excluding one sample (ST5 station, PA fraction, ES0121 cruise) due to low sequencing depth (913 reads). Alpha diversity was assessed using the Chao1 richness index (as singletons were retained in the dataset) and the Shannon diversity index [41]. Beta diversity was evaluated with Bray–Curtis dissimilarities on square root-transformed, normalized data, visualized via non-metric multidimensional scaling (NMDS). Alpha and beta diversity analyses were conducted with the “vegan” package [42]. Environmental variable correlations with community ordination were tested using envfit, from the “vegan” package [42]. The Particle-Associated Niche (PAN) index [43] was computed using the “EcolUtils” package [44] to estimate ASV association with the particle-associated fraction based on their relative abundances. A Coast-Offshore Niche (CON) index was similarly developed to quantify ASV preferences along the coast-to-offshore gradient. The minimum distance of each sampling station to the coastline was used as the environmental variable (“env.var”) in the niche.val() function implemented in the “EcolUtils” package [44]. Distances to the coastline were calculated using the “rgeos” package [45], based on shapefiles from the European Environment Agency and spatial projections handled via “rgdal” [46]. Spearman correlations between dominant phylogroups (>2% of mean relative abundance) and environmental variables were computed using the “hmisc” package [47]. The same environmental data were used for both fractions. *P*-values were adjusted using the Bonferroni correction [48]. Additionally, phylogroups were clustered by environmental variables using the “pheatmap” package [49]. Differences in microbial abundances between size fractions and seasons were evaluated by one-way ANOVA after testing for normality (Kolmogorov–Smirnov) and homoscedasticity (Levene’s test) using the “car” package [50, 51]. When necessary, data were transformed to meet assumptions required for one-way ANOVA [52]. When transformations were insufficient, the Kruskal–Wallis test was used [53]. *Post hoc* tests included Tukey’s test for one-way ANOVA [54] and Dunn’s test for Kruskal–Wallis [55].

## Results

### Environmental context

Sea surface temperature (SST) ranged from 13.9 to 23.3°C (mean ± SD, 15.9 ± 1.8°C) across all cruises (Fig. S1, Table S2), with the highest values recorded during the spring cruise ES0620 (20.73 ± 1.68°C, *P* < .05). Sea surface salinity (SSS) ranged from 34.7 to 37.9 (36.7 ± 0.6), being significantly lower during the winter cruises ES0121 and ES0122 and the spring cruise ES0322 (36.17 ± 1.16, *P* < .05). When comparing transects, the AL transect showed significantly higher salinity than the other transects (37.07 ± 0.33, *P* < .05).

Dissolved oxygen (DO) ranged from 115.9 to 287.6 μM (232.4 ± 45.0 μM), with significantly lower values observed during the autumn cruise ES1122 (157.2 ± 12.4 μM) compared to all other cruises (*P* < .05), except ES0620. Total chlorophyll *a* (Chl *a*) and nutrient concentrations (nitrate, nitrite, phosphate, and silicate) were significantly higher during cruise ES1122 (*P* < .05). Specifically, mean ± SD concentrations during this cruise were as follows: Chl *a*, 2.02 ± 1.84 μg L^−1^; NO₃^−^, 6.22 ± 2.79 μM; NO₂^−^, 0.45 ± 0.09 μM; PO₄^3−^, 0.52 ± 0.09 μM; and SiOH, 4.00 ± 1.84 μM. The combination of elevated SSS, total Chl *a*, and nutrient concentrations, along with lower SST and DO values, suggests an upwelling event occurred during this cruise. Ammonium (NH₄^+^) concentrations ranged from below detection limit to 5.38 μM (0.56 ± 0.45 μM), with significantly higher concentrations recorded during winter cruises ES0120 and ES0121, as well as autumn cruise ES1122 (*P* < .05), with a mean value of 0.77 ± 0.72 μM for these three cruises. Among individual stations within transects, only SiOH concentrations at ST1 were significantly higher than at other ST stations (*P* > .05; Table S2).

### Enumeration of AAP bacteria and other microbial groups

AAPs exhibited significantly higher absolute (2.92·10^4^ ± 2.07·10^4^ cells ml^−1^) and relative abundances (4.24 ± 3.07% of total heterotrophic bacteria) during the spring cruise ES0521 (Fig. S2, Table S3), compared to winter cruises ES0121 and ES0122 (*P* < .05). *Synechococcus* abundances (1.43·10^4^ ± 1.32·10^4^ cells ml^−1^) were higher (*P* < .05) during cruises ES0521, ES0120, ES0620, and ES1122 than in cruises ES0121, ES0122, and ES0322. In contrast, *Prochlorococcus* abundances were significantly higher (*P* < .05) during winter and autumn cruises ES0120, ES0121, and ES1122 (5.14·10^3^ ± 6.09·10^4^ cells ml^−1^), as were photosynthetic picoeukaryotes (PPEs) during cruises ES0120, ES0122, and ES1122 (1.39·10^4^ ± 8.70·10^3^ ml^−1^). No significant seasonal differences were detected for total bacterial abundance or low (LNA) and high nucleic acid (HNA) bacteria.

Spatially, AAP absolute abundance was highest along the MA transect (3.91·10^4^ ± 2.38·10^4^ cells ml^−1^), while relative abundance peaked in AL (5.68 ± 4.66%). In contrast, both AAP metrics were lowest along the AG transect. Similarly, *Synechococcus* abundances peaked in AL (2.01·10^4^ ± 1.58·10^4^ cells ml^−1^), being significantly higher (*P* < .05) than in MA and AG. Conversely, *Prochlorococcus* abundances varied significantly among the ST, MA, and AL transects (*P* < .05), with the highest values recorded in ST (7.60·10^3^ ± 8.53·10^3^ cells ml^−1^). PPE abundances were significantly lower in AL compared to ST and MA (*P* < .05), while total heterotrophic bacteria, LNA, and HNA abundances showed no significant differences among transects.

No consistent patterns in AAP absolute or relative percentage were observed across stations within each transect. However, AAP percentages were generally lower at coastal stations (Table S2), with a similar trend for absolute abundance. Neither AG nor ST showed any clear AAP spatial trends. Interestingly, in ST and MA, both with greater offshore extent, *Synechococcus* and *Prochlorococcus* abundances increased with distance from the coast, along with the AAP percentage in MA (Table S3). In contrast, total bacterial, LNA, and HNA abundances were generally higher at coastal stations in AL and MA, while in ST, these groups peaked at the mid-shelf station ST5 (Table S3).

### A‌AP community composition differs across space and time

Of the 221 samples from which DNA was extracted, 26 particle-associated (PA) samples failed to amplify the *pufM* gene, resulting in a final dataset of 195 samples with successful amplification and high-quality sequences (Table S1). To investigate the cause of these failures, we examined differences in DNA concentration and found significant variation between the two fractions (FL and PA; *P* < .001) as well as between successfully amplified and failed samples (Fig. S3A). This pattern persisted when analyzing only the PA fraction (*P* < .05) and when considering both fractions together (*P* < .001). Additionally, within the PA fraction specifically, failed samples also showed significantly lower AAP concentrations (Fig. S3B, *P* < .01) and lower AAP proportions (Fig. S3C, *P* < .05) compared with samples that amplified successfully.

We classified ASVs into 14 taxonomic groups and explored their spatial and temporal patterns across FL and PA fractions. The most prevalent groups were phylogroups A, K, Rhodobacterales G1, and B (Fig. S4), with their relative proportions varying among cruises, transects, and distance from the coast. The Pseudomonadales group K exhibited significantly higher relative abundance (*P* < .001) during late spring (cruises ES0521 and ES0620; 38.9% mean), compared to early spring and winter (cruises ES0121, ES0122, and ES0322; 16.6% mean). This group was consistently less abundant at offshore stations. In contrast, uncultured phylogroups A and B dominated during early spring and winter (cruises ES0120, ES0121, ES0122, and ES0322; 26.1% combined mean), with higher relative abundances at offshore stations (e.g. ES0122 ST7 FL and PA, ES0322 MA5 PA, or ES1122 AL5 FL), where their combined mean abundance reached 22.2% (Fig. S4). In comparison, their relative abundances decreased during certain spring (ES0521) and autumn (ES1122) cruises, with a combined mean of 13.8%.

Among transects, Rhodobacterales G1 showed significantly higher relative abundance in AG (mean relative abundance: 24.7%; *P* < .001) (Fig. S4). In other transects, high Rhodobacterales G1 abundances were generally observed at coastal stations (e.g. ES0121 MA2 and ES1122 AL1) with a mean abundance ~30%, mirroring the distribution of Pseudomonadales group K. Furthermore, certain phylogroups displayed higher relative abundances during specific cruises; Burkholderiales were more prevalent in cruise ES0322 (mean: 2.8%; *P* < .05), while the uncultured phylogroup C peaked in cruises ES1122 and ES0120 (mean: 11.3%; *P* < .05).

A detailed analysis of ASV relative abundances revealed that the 10 most abundant variants belonged to phylogroups A, B, C, K, and Rhodobacterales G1 (Fig. S5). Among these, dominant ASV1 (phylogroup A) displayed strong temporal patterns, being most prevalent during winter cruises ES0120, ES0121, and ES0122, peaking in ES0322 (22.1% mean relative abundance) but remaining below 10% along the AG transect. Similarly, ASV10 (phylogroup B) showed a clear winter preference and was least represented along the AG transect. In contrast, the most abundant phylogroup K ASVs (ASV2, ASV3, ASV5) peaked during the non-winter cruises ES0521, ES0620, and ES1122, reflecting a seasonal shift in community composition. Notably, ASV14, also belonging to phylogroup K, deviated from this pattern by reaching its highest relative abundance during the winter cruise ES0120 (mean: 5.6%). Distinct from these temporal patterns, ASVs affiliated with Rhodobacterales G1 (ASV4 and ASV7) showed a marked spatial preference, consistently being more prevalent along the AG transect (mean: 5.4%), suggesting a preference for local environmental conditions. Interestingly, a few samples, particularly in the PA fraction, exhibited a markedly different taxonomic composition, for example the PA sample AL2 from ES0322 cruise. In particular, the AAP community in this sample differed substantially from all other samples. Phylogroups I and J reached 24.2% and 20.2% relative abundance, respectively, the highest proportions observed across the dataset, while other typically minor groups, such as Rhizobiales (14.2%) and Sphingomonadales (10.0%), also appeared at unusually elevated levels.

### Particle association enhances diversity in AAP bacteria

**Figure 2:**
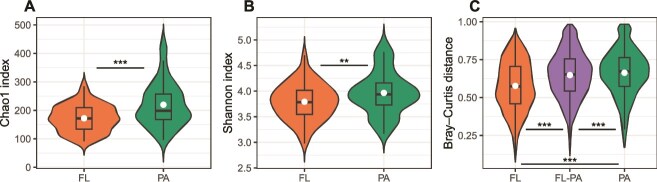
Diversity of AAP communities in the free-living (FL) and particle-associated (PA) fractions. (A) Chao1 richness. (B) Shannon diversity indices. (C) Bray–Curtis dissimilarity indices comparing community composition within FL, between FL and PA (FL-PA), and within PA communities. White dots represent mean values. Statistical significance (Kruskal-Wallis): ^*^P < .05, ^**^P < .01, ^***^P < .001.

**Figure 3:**
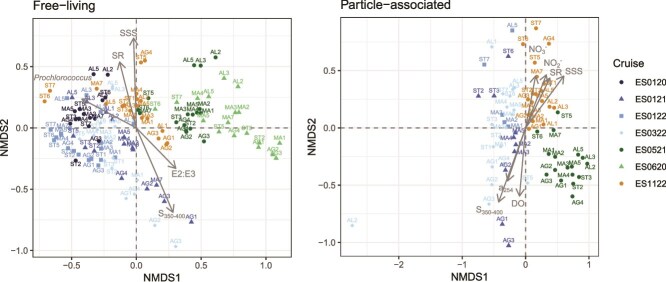
Non-metric multidimensional scaling (NMDS) ordinations of AAP communities in the free-living (left) and particle-associated (right) fractions. Points are shaded by cruise season and shaped by cruise year. Sampling stations are indicated by labels. Arrows represent significant continuous environmental variables (*P* < .05) from the environmental fitting analysis (envfit). Variables include *Prochlorococcus* abundance [*Prochlorococcus*], sea surface salinity [SSS], dissolved oxygen [DO], nitrate [NO^₃−^], nitrite [NO^₂−^], absorbance coefficient at 254 nm [*a*_254_], absorbance ratio 250/365 nm [E2:E3], logarithmic regression slope for absorbance coefficients between 350 and 400 nm [*S*_350–400_], and the ratio between *S*_275–295_ and *S*_350–400_ [SR].

To study the diversity of AAP bacteria, alpha diversity was assessed with the Chao1 and Shannon indexes, while beta diversity was analyzed with Bray–Curtis distances. These indices revealed that PA communities were significantly richer (Chao 1, *P* < .001) and more diverse (Shannon, *P* < .01) than FL communities (Fig. 2A, B). Alpha-diversity indices in the two fractions were strongly and positively correlated (Chao1, *n* = 69, Pearson correlation *R*^2^ = 0.50, *P* < .001; Shannon, *n* = 68, Pearson correlation *R*^2^ = 0.64, *P* < .001). To further investigate differences between lifestyles, we examined ASVs unique to each fraction. Out of the final 6930 ASVs, 4471 (~65%) were exclusive to PA samples; however, they were mostly rare, accounting for only 2% of the *pufM* gene reads. In contrast, ~97.5% of *pufM* reads were represented by the 1341 shared ASVs. Beta diversity revealed that distances between FL and PA communities (FL–PA), as well as among PA communities themselves, were significantly greater (*P* < .001, Fig. 2C) than those among FL communities. Moreover, distances among PA communities were also significantly greater (*P* < .001) than distances between FL and PA communities. NMDS plots for each fraction revealed consistent seasonal patterns (Fig. 3); samples collected during winter and early spring cruises were more similar among each other than to those from late spring, which also grouped closely. Autumn samples from cruise ES1122 formed a distinct transitional group between winter (ES0120, ES0121, ES0122, ES0322) and spring samples (ES0521, ES0620). “Cruise” had a significant effect on community structure in both fractions (FL: *R*^2^ = 0.34; PA: *R*^2^ = 0.58). Additionally, “Season” significantly influenced the PA community (*R*^2^ = 0.57), indicating strong temporal structuring across both communities. In the FL fraction, few variables were strongly correlated with community structure (*R*^2^ > 0.5), with *S*_350–400_ showing the strongest correlation, followed by salinity, the ratio between *S*_275–295_ and *S*_350–400_, *Prochlorococcus* abundance, and the absorbance ratio between 250 and 365 nm (Table S4). These environmental factors contributed to clear spatial and seasonal patterns: SSS and SR were associated with ES1122 samples, likely reflecting upwelling; *Prochlorococcus* aligned with AL stations; and other CDOM proxies (*S*_350–400_, E2:E3) were linked to AG stations and early spring samples. In the PA fraction, community structure was influenced by CDOM proxies (*S*_350–400_, *a*_254_, SR), SSS, nitrate (NO_3_^−^), nitrite (NO₂^−^), and DO. As in the FL fraction, SSS and SR correlated with ES1122 samples. However, in the PA fraction ES1122 samples also correlated with nitrate and nitrite. Again, CDOM variables (*S*_350–400_, *a*_254_) were linked to AG stations during early spring cruises (ES0121, ES0322), along with DO.

### Distinct AAP phylogroups exhibit niche preference

**Figure 4:**
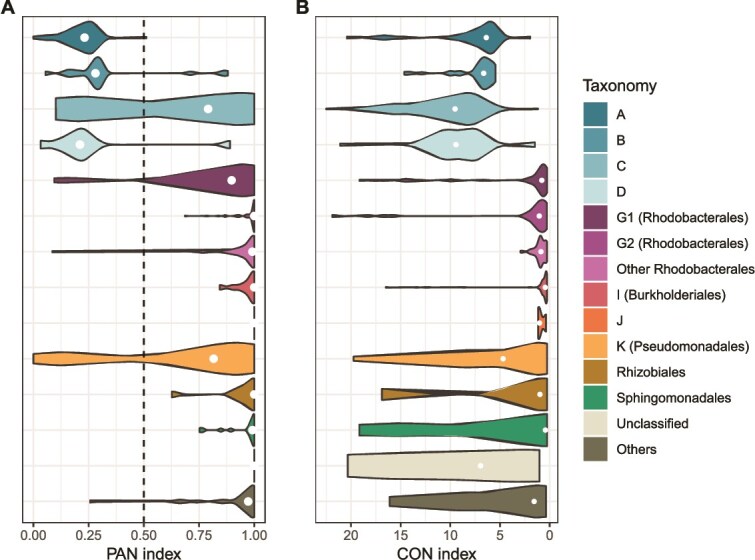
(A) Particle-associated niche (PAN) index values of the amplicon sequence variants (ASVs) showing significant (*P* < .05) lifestyle preferences, grouped by taxonomic affiliation. PAN values close to 0 indicate a preference for free-living (FL) and near 1 for particle-associated (PA) lifestyle. (B) Coast-Offshore Niche (CON) indices of the ASVs with significant (*P* < .05) spatial preferences. Lower CON values indicate coastal preference, while higher values indicate offshore preference. White dots represent the mean PAN or CON index for each phylogroup.

Differences in AAP phylogroup preferences between the two fractions were evaluated by statistically comparing their relative abundances. At the phylogroup level, AAP community composition differed significantly between FL and PA fractions (*P* < .05; Fig. S4, Table S5). Phylogroups K (Pseudomonadales) and I (Burkholderiales) were consistently more abundant in PA, whereas alphaproteobacterial groups, such as phylogroup J (Rhizobiales) and Sphingomonadales, were less represented. Conversely, uncultured phylogroups A, B, C, and D were significantly enriched in the FL fraction (*P* < .05). Overall, the top 10 ASVs of the entire dataset showed minimal differences between FL and PA fractions (Fig. S5). ASV1 (phylogroup A) displayed a slightly higher abundance in the FL fraction (15.9%) compared to the PA fraction (9.9%), whereas most phylogroup K ASVs (e.g. ASV2, ASV3, and ASV5) were more abundant in the PA fraction. Interestingly, the order Rhodobacterales showed contrasting lifestyle distributions: Rhodobacterales G1 was more common in the FL fraction, whereas Rhodobacterales G2 and “Other Rhodobacterales” were enriched in the PA communities (*P* < .05). Within the FL fraction, the dominant Rhodobacterales genera included *Roseovarius* (ASV102, ASV315), *Jannaschia* (ASV553), *Roseobacter* (ASV880), *Sulfitobacter* (ASV34), and *Thalassobacter* (ASV288). In PA samples, the prevailing genera were *Roseivivax* (ASV162), *Dinoroseobacter* (ASV310), *Tateyamaria* (ASV368), and *Yoonia* (ASV195). To investigate ecological preferences among AAP taxa, we calculated the PAN index for each ASV (Fig. 4A), yielding significant results (*P* < .05) for 372 of the 6927 ASVs. Preferences were stronger in the PA fraction, with 262 ASVs (70.4%) scoring above 0.50 in the PAN index. In contrast, uncultured phylogroups A, B, and D were predominantly associated with FL communities, as 87.5% of their ASVs displayed PAN values below 0.30. Among the less abundant phylogroups (Rhodobacterales G2, “Other Rhodobacterales”, I and J, Rhizobiales, Sphingomonadales, “Unclassified”, and “Others”), 93.9% of their ASVs had PAN values above 0.75 (PA preference). Notably, phylogroups C, K, and Rhodobacterales G1 showed broader niche ranges, with ASVs spanning preference for both fractions (PAN values both above or below 0.50).

Additionally, we developed the CON index to explore spatial preferences (Fig. 4B). Out of the 6927 ASVs, 537 (7.75% of total ASVs) showed significant spatial preference (*P* < .05). Roughly half preferred coastal habitats (*n* = 259), and the other half offshore (*n* = 278), using a 5-nautical-mile threshold to distinguish coastal from offshore environments. Rhodobacterales and phylogroups I, J, and Rhizobiales showed strong coastal associations (90.9% ASVs with CON index <5). In contrast, uncultured phylogroups A, B, C, and D consistently preferred offshore habitats (CON index >5). Other phylogroups were more variable: phylogroup K had a mean CON index of 6.2 with 8.3% of its ASVs scoring >15, while Sphingomonadales showed a coastal tendency (median 0.4, mean 5.4) but included offshore outliers (~20% of ASVs scoring >15). A total of 111 ASVs were significant for both PAN and CON indices; among these, 47 ASVs (PAN index <0.5, CON index >5) were associated with free-living, offshore niches, while 45 ASVs (PAN index >0.5, CON index <5) were linked to particle-associated, coastal environments.

### Differential environmental influence on FL and PA fractions

**Figure 5:**
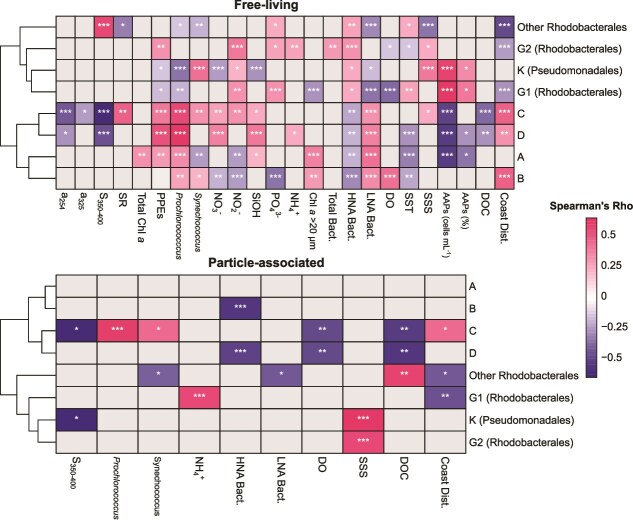
Heatmaps of Spearman rank correlation coefficients (ρ) between the relative abundance of dominant aerobic anoxygenic phototrophic (AAP) bacterial phylogroups (relative abundance >1%) and environmental variables showing significant correlations (*P* < .05). Results are shown for the free-living (FL) fraction (top) and the particle-associated (PA) fraction (bottom). Axes are clustered to improve visualization. Asterisks denote significance levels after Bonferroni correction (^*^P < .05, ^**^P < .01, ^***^P < .001), while gray cells indicate no significant correlations (*P* > .05). Environmental variables include distance to the coast [coast Dist.], AAP abundance [AAPs], AAP percentage relative to total bacteria [AAPs (%)], total bacterial abundance [total Bact.], high-nucleic acid bacteria [HNA Bact.], low-nucleic acid bacteria [LNA Bact.], *Synechococcus* abundance, *Prochlorococcus* abundance, pigmented picoeukaryote abundance [PPEs], sea surface salinity [SSS], total chlorophyll *a* [total Chl *a*], chlorophyll *a* in the fraction greater than 20 μm [Chl *a* > 20 μm], dissolved oxygen [DO], nitrate [NO^−^₃], nitrite [NO^−^₂], phosphate [PO^3−^₄], silicate [SiOH], ammonium [NH^+^_4_], dissolved organic carbon [DOC], absorbance coefficient at 254 nm [*a*_254_], absorbance coefficient at 325 nm [*a*_325_], logarithmic regression slope for absorbance coefficients between 350 and 400 nm [*S*_350–400_], and the ratio between *S*_275–295_ and *S*_350–400_ [SR].

To identify environmental drivers shaping AAP communities, Spearman correlation coefficients were calculated between the relative abundances of dominant phylogroups and environmental variables (Fig. 5). Hierarchical clustering of these correlations revealed two major clusters in both fractions. Members of the order Rhodobacterales and phylogroup K formed a cluster that positively correlated with nutrients (NO_2_^−^, NH_4_^+^, PO_4_^3−^), AAP abundance, HNA bacteria, DOC, and salinity in both fractions. These taxa also showed negative correlations with *Prochlorococcus* abundance, LNA bacteria, and distance from the coast in both fractions. The second major cluster comprised uncultured phylogroups A, B, C, and D, which consistently formed two subclusters: A–B and C–D. Uncultured phylogroups were consistently associated with offshore conditions, showing positive correlations with distance from the coast and *Prochlorococcus* abundance. In contrast, they were negatively correlated with AAP and HNA bacterial abundances, as well as with DOC and CDOM variables.

## Discussion

The Alboran Sea is a transitional basin between the Atlantic Ocean and the Mediterranean Sea, where the inflow of Atlantic water through the Strait of Gibraltar generates distinctive mesoscale structures, including two large anticyclonic gyres and upwelling zones along the northern coast. These structures vary seasonally: strong stratification dominates in summer, while winter deep mixing reshapes physical and biological conditions [56, 57]. Resulting environmental gradients influence microbial community distribution and activity [58–60]. Yet, aerobic anoxygenic phototrophic (AAPs) in this region remained largely uncharacterized. The cruises conducted in this study encompass broad environmental variability, capturing seasonal transitions, coast-to-offshore gradients, and episodic upwelling events, providing a comprehensive framework to examine the drivers of microbial assemblages.

Overall, the relative abundance of AAPs ranged from 0.62% to 13.9% (mean 4.34%), being these percentages and the recorded cell abundances in line with previous reports in other Mediterranean areas [3, 13, 17] and higher than in open ocean waters [5, 61]. Although the amount of microscopy data in our study is limited, higher values were recorded in spring (ES0521) compared to winter (ES0121, ES0122), a pattern previously observed in the NW Mediterranean [11, 62]. These higher abundances in cruise ES0521 coincided with higher Chl *a* concentration, lower nutrient levels (NO₃^−^, PO₄^3−^, SiOH), and increased *Synechococcus* abundance, suggesting a link between AAP dynamics and primary production. Longer daylight increases photosynthesis, which reduces dissolved nutrients and enhances the release of DOM from phytoplankton, conditions that may favor the growth of AAP bacteria [27, 63, 64]. Altogether, the abundance patterns observed in this study align with previous observations showing a close ecological association between AAP bacteria and phytoplankton communities [5, 11, 65, 66].

AAP community composition exhibited clear spatial and temporal patterns of diversity. AAP assemblages were taxonomically diverse, but consistently dominated by Alphaproteobacteria, particularly by members of the Rhodobacteraceae family, along with uncultured phylogroups A and B, which were especially prominent during the cooler months (e.g. winter and early spring cruises), while members of the Gammaproteobacteria, notably Pseudomonadales (phylogroup K), became more abundant during warmer periods (spring cruises). Phylogroup K, affiliated to the NOR5/OM60 clade, has traditionally been considered among the most abundant components of AAP communities, particularly in the Mediterranean Sea [10, 11, 13]. In this study, we used primers with broader phylogenetic coverage [20], which captured the diversity of previously underrepresented groups, including uncultured phylogroups A, B, and C. These groups were predominant in the Alboran Sea, aligning with results obtained from primer bias-free metagenomics elsewhere [20].

Seasonality is a major driver of AAP community composition in aquatic ecosystems [11, 18, 64]. In our work, temporal factors (Cruise and Season) were the strongest predictors of AAP structure, with winter and spring communities being particularly distinct (Fig. 3), consistent with other observations across the Mediterranean Sea [11, 18]. Notably, winter conditions were characterized by reduced Rhodobacterales and Pseudomonadales relative abundance and increased phylogroups A, B, C, and D, in line with previous reports from the Western Mediterranean, showing winter dominance of phylogroups A and B [20] and year-round prevalence of Rhodobacterales and Pseudomonadales [11, 20]. These findings reinforce the strong seasonal imprint on AAP community structure and abundance.

Beyond characterizing spatiotemporal patterns, a central objective of this study was to compare free-living (FL) and particle-associated (PA) AAP communities. Although substantial compositional differences between FL and PA prokaryotic assemblages have been documented [36, 67–70], investigations targeting AAPs remain scarce. A challenge encountered in this study was the failure to amplify the *pufM* gene in 26 of the 98 PA samples. These samples were characterized by lower DNA concentrations and lower AAP abundances, suggesting that insufficient template availability likely contributed to the amplification failures. In addition, the use of larger-pore filters (3-μm *vs.* 0.2-μm) may have worsened the issue. Although larger pores are intended to capture particle-associated cells, they can also retain substantial amounts of particulate material that often contains PCR inhibitors such as salts, detritus, and organic compounds (e.g. fulvic and humic acids). The accumulation of these substances can interfere with DNA extraction and reduce amplification efficiency [71].

Despite these methodological limitations, our results reveal clear lifestyle-dependent differences, with higher diversity and greater specialization in the PA fraction (Fig. 2). In agreement, a recently published study [18] showed that the expression of phototrophy genes in AAP is also heterogeneous across seasons and fractions, with the PA community containing more active AAP bacteria. However, it is important to note that our study is based on DNA and therefore reflects the presence of phototrophy-related genes rather than their expression. Without RNA or pigment data, we cannot determine whether AAPs were actively performing phototrophy at the time of sampling. As a result, the strong association of particle-associated AAP communities with DOC, CDOM proxies, and particle-rich environments we observed likely reflect their heterotrophic ecological strategies rather than active phototrophy. This is consistent with previous work showing that organic matter availability often plays a more decisive role than light in shaping AAP abundance and composition [61].

Alpha-diversity analyses revealed that PA communities were significantly richer than FL communities, in contrast to the findings of Cottrell *et al*. [16]. Using denaturing gradient gel electrophoresis (DGGE) fingerprinting, those authors reported no diversity differences between the whole AAP community and the free-living fraction in the Chesapeake Bay, suggesting that lifestyle partitioning may depend on ecosystem characteristics (estuarine vs. coastal Mediterranean) or methodological choices. In our study, the PA fraction was defined using a 3-μm filter, whereas Cottrell *et al*. [16] used a 0.8-μm cutoff. Because many AAP cells are relatively large [72], a 0.8-μm filter likely retains a substantial proportion of free-living cells, reducing the contrast between fractions and potentially masking diversity differences. Moreover, DGGE offers much lower taxonomic resolution than high-throughput amplicon sequencing, detecting only dominant bands and grouping multiple taxa into single operational units. In contrast, our sequencing approach resolves hundreds of ASVs, enabling finer discrimination between FL and PA assemblages. Taken together, these methodological differences likely explain why we observe clear diversity differences between lifestyles, whereas earlier DGGE-based studies did not [16].

In addition to higher diversity, the PA fraction has been shown to harbor greater AAP abundances [17, 36, 73, 74] and more active AAPs, as shown recently in the Adriatic Sea [18]. Although we do not have fractionated abundance data, the dominant particle-associated taxa (Rhodobacterales and Pseudomonadales) were more prevalent in coastal stations, where total AAP abundance was generally higher. For instance, in the five samples with the highest AAP abundance, these phylogroups accounted for 69%–79% of the total *pufM* gene reads (Rhodobacterales: 21%–22%; Pseudomonadales: 48%–57%). In these particulate-rich coastal environments, accessible organic matter, protection from predation, and light modulation via particle backscattering may promote AAP proliferation [16], consistent with the coastal preference of most AAP phylogroups. The PA fraction also contained more unique ASVs, indicating that particles support more exclusive assemblages, likely due to their heterogeneity [75] and the ecological advantages they offer, such as protection from environmental stress and enhanced nutrient availability [68]. This habitat heterogeneity may explain the higher Bray–Curtis dissimilarities observed in this study among PA AAP communities compared to FL communities. Although higher FL diversity has occasionally been reported for the total prokaryotic community [43, 76, 77], larger size fractions typically host more ecological niches and, consequently, greater diversity within the total prokaryotic community [76–79]. Conversely, uncultured phylogroups A, B, C, and D were primarily associated with free-living (FL) communities in offshore oligotrophic waters. Their ecology remains poorly understood, in part due to primer biases that limit their detection in PCR-based studies [20]. Nevertheless, metagenomic data from the Global Ocean Sampling Expedition reported phylogroups A and B as abundant in oligotrophic environments [21]. In our phylogenetic reconstruction, sequences affiliated with phylogroups C and D cluster with members of the *Candidatus* Luxescamonaceae derived from single amplified genomes and metagenomic assembled genomes generated during the *Tara* Oceans expedition [37, 80]. This lineage has been hypothesized to perform CO₂ fixation [80] and was recently incorporated into the *Roseobacter* LUX cluster [81]. In particular, some members of phylogroups C might be related to the subcluster LUX-A, while phylogroup D may be related to subcluster LUX-I [81, 82]. Genome-centric analyses indicate that LUX members vary substantially in genome size, G + C content, and number of coding DNA sequences (CDS), raising questions about their ecological coherence. Nonetheless, our results suggest that phylogroups C and D, together with uncultured phylogroups A and B, seem to be adapted to low-nutrient, low-particle offshore environments. In concordance, several GTDB-derived *pufM* gene sequences assigned to the genera CAIJG01 and CACIIZ01 appear to be associated in our tree with phylogroups C and D, respectively, while sequences affiliated with the genus *Salinivens* cluster with phylogroups A and B. These three genera have been linked to marine, low-nutrient lifestyles in the Adriatic Sea [83] and Moreton Bay at East Australia [84]. In contrast, coastal samples in our study exhibited a higher prevalence of Rhodobacterales, Rhizobiales, and Sphingomonadales, consistent with previous studies associating Rhodobacterales (e.g. phylogroup G) with mesotrophic conditions [21].

Although AAP abundances by phylogroup or fraction were not directly quantified, the high relative abundances of copiotrophic taxa such as Rhodobacterales and Pseudomonadales in PA fractions during productive seasons and at coastal stations suggest enhanced growth under nutrient-rich conditions. These groups display high growth rates and respond rapidly to nutrient inputs [85, 86], and their positive correlations with total AAP abundance, HNA bacteria, and other productivity indicators align with the typically faster growth of HNA bacteria in eutrophic environments [87, 88]. Conversely, uncultured oligotrophic-associated phylogroups exhibited negative correlations with variables linked to organic matter concentration and lability (*a*_254_, *a*_325_, *S*_350–400_, DOC). The strong negative correlations of phylogroups C and D with DOM quality proxies suggest that these taxa are adapted to low organic matter availability and more recalcitrant carbon sources, characteristic of oligotrophic waters [89, 90]. Lower salinity appears to favor taxa associated with brackish or freshwater environments [91, 92]. In agreement, salinity had a role in modulating AAP communities, particularly during cruises ES0121 and ES0322, when the relative abundance of phylogroups I, J, and Rhizobiales increased in several PA samples. However, no seasonal pattern was evident in salinity or other oceanographic variables, likely due to water-mass stratification or mixing. Only one upwelling event was detected, during the ES1122 cruise, which likely explains the elevated salinity values resulting from the intrusion of subsurface Mediterranean water (salinity ~37.5), as well as the concurrent increases in nitrate and silicate concentrations [57].

## Conclusions

This study reveals pronounced seasonal and spatial structuring of aerobic anoxygenic phototrophic (AAP) bacterial communities in the Alboran Sea. Coastal spring communities were dominated by Rhodobacterales and Pseudomonadales, whereas uncultured phylogroups prevailed in winter offshore waters. These shifts were tightly linked to environmental variability (including salinity, nutrients, CDOM) which together shaped the seasonal and coast-to-offshore dynamics of AAP assemblages. Clear lifestyle-dependent differences were also observed. Particle-associated communities exhibited higher alpha diversity, a larger number of unique ASVs, and a broader phylogroup representation, likely reflecting the heterogeneous microenvironments and resource gradients provided by particles. In contrast, free-living assemblages were compositionally more constrained, dominated by uncultured phylogroups A, B, C, and D, particularly under oligotrophic offshore conditions. Although these groups remain poorly understood, our findings underscore their ecological relevance as specialists in low-nutrient, particle-poor environments. Overall, lifestyle differentiation emerged as a key factor structuring AAP communities in the Alboran Sea, reflecting distinct ecological niches and resource partitioning shaped by habitat heterogeneity and environmental forcing across the basin.

## Supplementary Material

ycag120_Supplementary_Materials_clean_AAPs_R2

## Data Availability

*pufM* amplicon sequences have been deposited in the NCBI Sequence Read Archive (SRA) under BioProject ID PRJNA1163263. Additional data and analysis scripts used in this work are publicly available in the study’s GitHub repository to ensure full reproducibility: https://github.com/jjsantosbruna/FL-PA_AAPs_AlboranSea_2026
