# Supplementary material for: Contrasting free-living and particle-associated aerobic anoxygenic phototrophic bacterial communities across space and time in the Alboran Sea
Source: ISME Commun. 2026 Apr 29;6(1):ycag120. doi: 10.1093/ismeco/ycag120 (PMC13219738; doi:10.1093/ismeco/ycag120)
Supplement: ycag120_Supplementary_Materials_clean_AAPs_R2 [file ycag120_supplementary_materials_clean_aaps_r2.docx]

**Supplementary Materials:**

**Contrasting free-living and particle-associated aerobic anoxygenic phototrophic bacterial communities across space and time in the Alboran Sea**

Jorge J. Santos-Bruña, Carlota R. Gazulla, Ana M. Cabello, Candela García-Gómez, Soluna Salles, M. Teresa Camarena-Gómez, Miriam Domínguez-Rodríguez, Antonio Sánchez, Nerea Valcárcel-Pérez, Francisco Gómez-Jakobsen, Seyed Mohammad Sadeghi-Nassaj, Isabel Reche, Barbara Marie, Eva Ortega-Retuerta, Lidia Yebra, Jesús M. Mercado, and Isabel Ferrera

**Supplementary Tables:** (For file download)

**Table S1.** Sample metadata with amplification, AAP Abundance, and DNA yield.

**Table S2.** Descriptive statistics of oceanographic variables by transect and sampling station.

**Table S3.** Descriptive statistics of surface microbial abundances by transect and sampling station.

**Table S4.** Descriptive statistics from the *envfit* analysis.

**Table S5**. Relative abundances (ab. %) by phylogroup.

**Supplementary Figures:**

**Fig. S1.** ​Boxplots of oceanographic variables by cruise.

**Fig. S2.** ​Boxplots of microbial abundances by cruise.

**Fig. S3.** Boxplots of DNA yield and AAP abundance by *pufM* gene amplification success.

**Fig. S4.** Relative contribution of AAP phylogroups to *pufM* gene reads by station and fraction.

**Fig. S5**. Bubble plots showing the relative abundance of the top 10 ASVs.

**Supplementary Information**

**SI1.** Epifluorescence microscopy methodology for AAP abundance.

**Supplementary Tables:**

**Table S1.** Overview of sample amplification success, AAP abundance, and DNA yield. Sample ID denotes the unique identifier of each sample; Cruise refers to the oceanographic expedition; Transect referes to the sampling transect; and Station indicates the corresponding sampling station. Filter Size (µm) indicates the pore size of the filter used (0.2 µm = free-living fraction, FL; 3 µm = particle-associated fraction, PA). QUBIT DNA (ng/µL) represents the DNA concentration measured by fluorometry. Amplified *pufM* gene indicates whether the *pufM* gene was successfully amplified by PCR; Processed *pufM* gene reads corresponds to the number of sequences reads obtained after bioinformatic processing (samples labelled “Not amplified” yielded no reads). AAPs (cells/mL) and AAPs (%) represent the abundance and relative proportion of aerobic anoxygenic phototrophic bacteria (AAPs), respectively; “Not measured” indicates that the parameter was not determined. *Table available for download.*

**Table S2.** Descriptive statistics of surface oceanographic variables by transect and sampling station. For each variable, the mean, standard deviation (SD), and range (minimum-maximum values in parentheses) are reported. Variables include: sea surface temperature [SST], sea surface salinity [SSS], total chlorophyll *a* [Total Chl a], dissolved oxygen [DO], nitrate [NO₃^-^], nitrite [NO₂^-^], ammonium [NH₄^+^], phosphate [PO₄^3-^], and silicate [SiOH] concentrations. *Table available for download.*


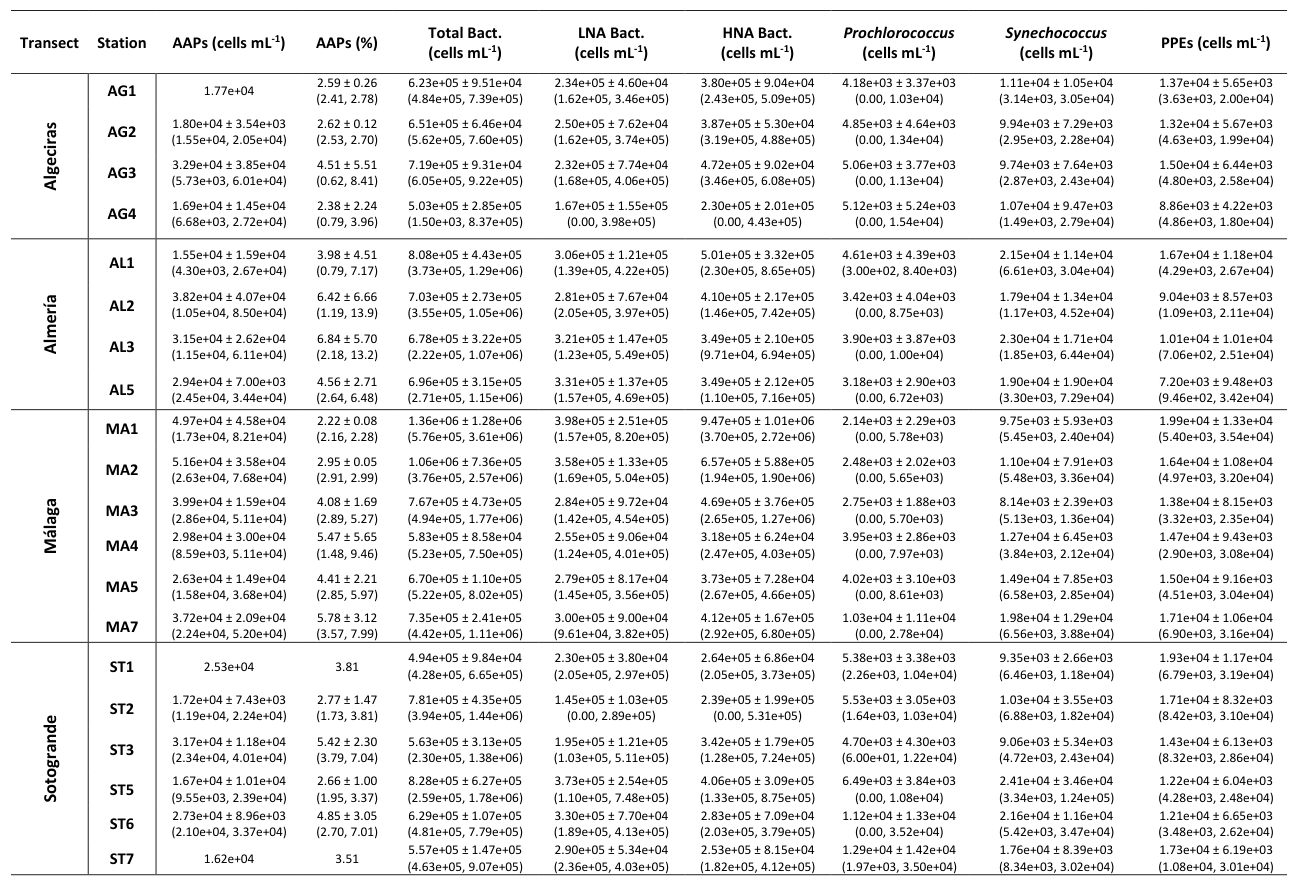
**Table S3.** Descriptive statistics of surface microbial abundances by transect and sampling station. The mean, standard deviation (SD), and range (minimum-maximum values in parentheses) are reported. A single value indicates that only one sample was collected (for AAPs data). Variables include: aerobic anoxygenic phototrophic bacteria [AAPs] abundance, percentage of AAPs relative to total bacteria [AAPs (%)], total bacterial abundance, Low Nucleic Acid [LNA] bacteria, High Nucleic Acid [HNA] bacteria, *Prochlorococcus* abundance, *Synechococcus* abundance, and pigmented picoeukaryote [PPEs] abundance. *Table available for download.*

**Table S4.** Descriptive statistics from the *envfit* analysis for both fractions (FL and PA) across categorical and continuous environmental factors. Missing values are indicated by ‘–‘. Statistically significant values (*p* < 0.05) are in blue and marked with an asterisk (*). Variables include: sea surface temperature [SST], sea surface salinity [SSS], total chlorophyll *a* [Total Chl *a*], chlorophyll *a* in the fraction greater than 20 µm [Chl *a*>20 µm], dissolved oxygen [DO], nitrate [NO₃^-^], nitrite [NO_2_^-^], ammonium [NH^4+^], phosphate [PO_4_^3-^], and silicate [SiOH] concentrations, aerobic anoxygenic bacteria [AAPs] abundance, percentage of AAPs relative to total bacteria [AAPs (%)], total bacterial abundance [Total Bact.], low nucleic acid bacteria [LNA Bact.], high nucleic acid bacteria [HNA Bact.], *Prochlorococcus*, *Synechococcus*, and pigmented picoeukaryote [PPEs] abundance, absorbance coefficient at 254 nm [a254], absorbance coefficient at 325 nm [a325], ratio between absorbance at 250 and 365 nm [E2:E3], ratio between absorbance at 465 and 665nm [E4:E6], logarithmic regression slope for absorbance coefficients between 350 and 400 nm [S_350-400_], logarithmic regression slope for absorbance coefficients between 275 and 295 nm [S_275-295_], and the ratio between S_275-295_ and S_350-400_ [SR].  *Table available for download.*

**Table S5**. Mean relative abundances (ab. %) of each AAP phylogroup for the overall composition (Total) separately by fraction (free-living, FL; particle-associated, PA), with *p*-values from Kruskal-Wallis tests comparing abundances between fractions (FL *vs* PA). Statistically significant differences (*p* < 0.05) are in blue and marked with an asterisk (*). Mean relative abundances (ab. %) were calculated as the mean of the relative abundances of each phylogroup across samples. For the mean relative abundance of a given fraction, only samples belonging to that fraction were considered. Statistical tests were performed using the relative abundances of each phylogroup in each individual sample, taking into account the fraction to which each sample belonged. The sample size for each statistical test corresponds to the number of samples successfully amplified in each fraction: 123 for FL and 72 for PA. *Table available for download.*


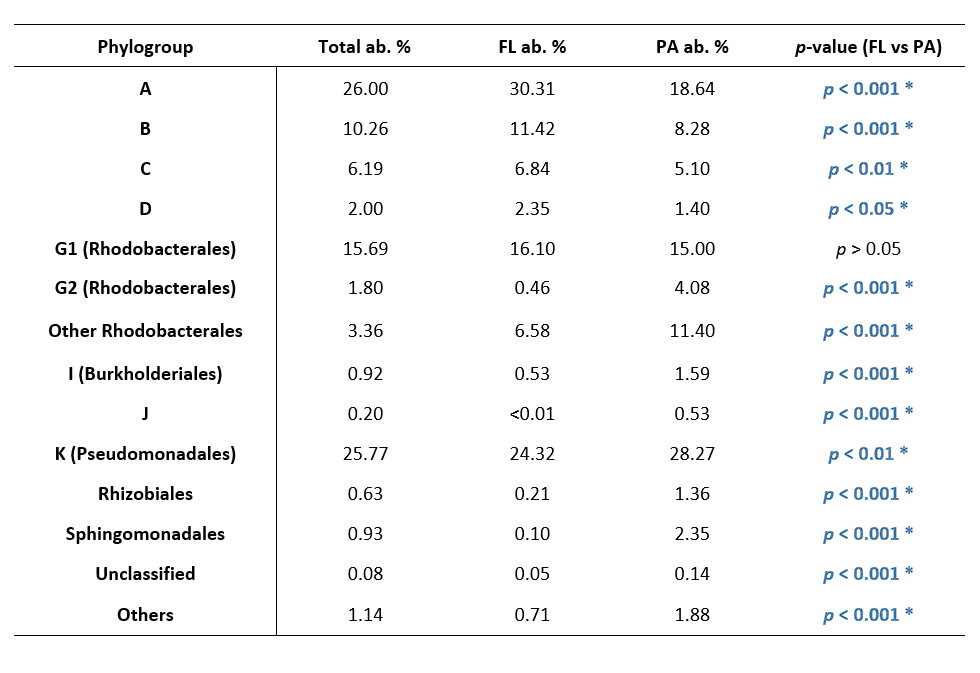


**Supplementary Figures:**


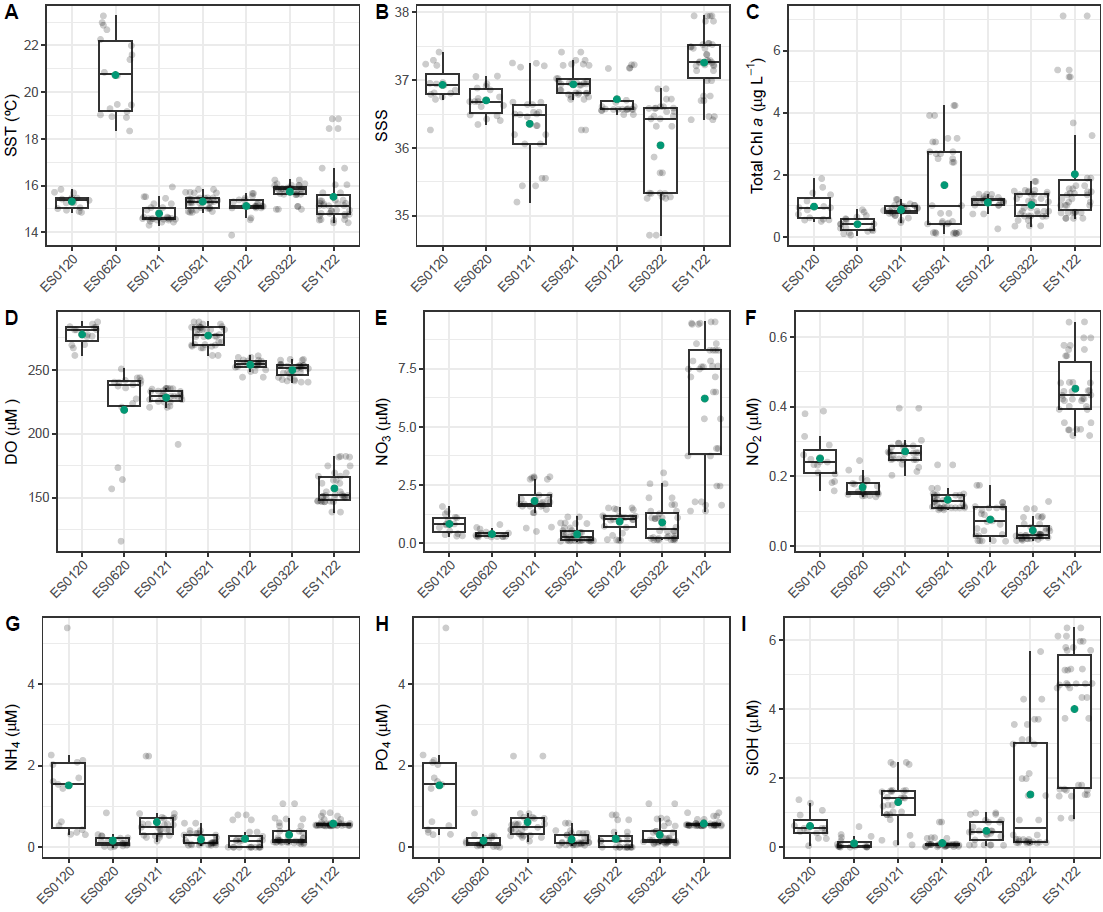


**Fig. S1.** ​Boxplots of surface oceanographic variables by cruise. Panels show: (A) sea surface temperature [SST], (B) sea surface salinity [SSS], (C) total chlorophyll *a* [Total Chl *a*], (D) dissolved oxygen [DO], (E) nitrate [NO₃^-^], (F) nitrite [NO₂^-^], (G) ammonium [NH₄^+^], (H) phosphate [PO₄^3-^], and (I) silicate [SiOH] concentrations. Individual samples are shown as grey dots, means as green dots, and medians by horizontal black lines.

**Fig. S2.** Boxplots of microbial abundances by cruise. Panels show (A) aerobic anoxygenic phototrophic bacteria [AAPs] abundance, (B) percentage of AAPs relative to total bacteria [AAPs (%)], (C) total bacterial abundance, (D) low nucleic acid [LNA] bacteria, (E) high nucleic acid [HNA] bacteria, (F) *Prochlorococcus* abundance, (G) *Synechococcus* abundance, and (H) photosynthetic picoeukaryote [PPEs] abundance. Individual samples are shown as grey dots, means as green dots, and medians as horizontal black lines.


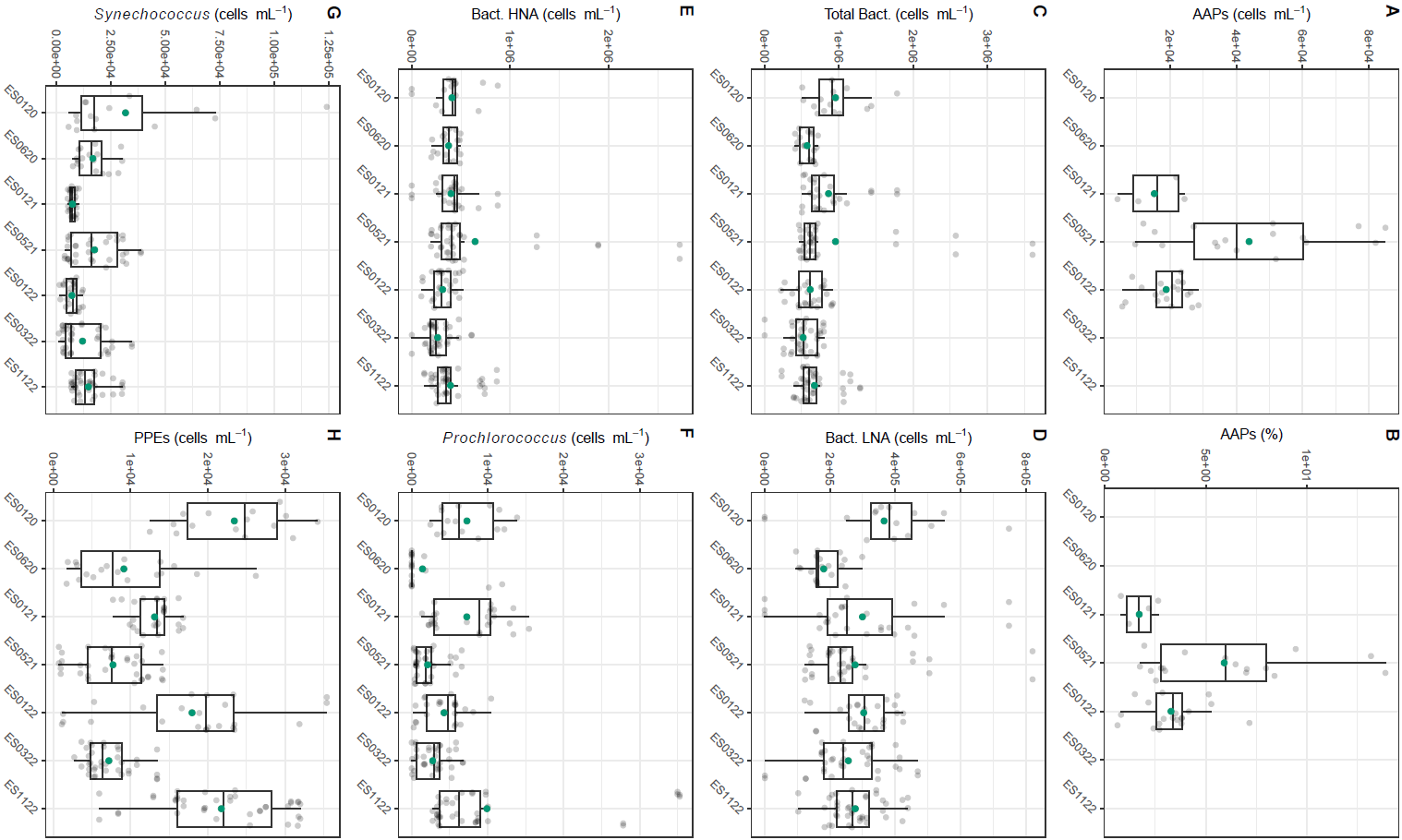


**Fig. S2.** Boxplots of microbial abundances by cruise. Panels show (A) aerobic anoxygenic phototrophic bacteria [AAPs] abundance, (B) percentage of AAPs relative to total bacteria [AAPs (%)], (C) total bacterial abundance, (D) low nucleic acid [LNA] bacteria, (E) high nucleic acid [HNA] bacteria, (F) *Prochlorococcus* abundance, (G) *Synechococcus* abundance, and (H) photosynthetic picoeukaryote [PPEs] abundance. Individual samples are shown as grey dots, means as green dots, and medians as horizontal black lines.


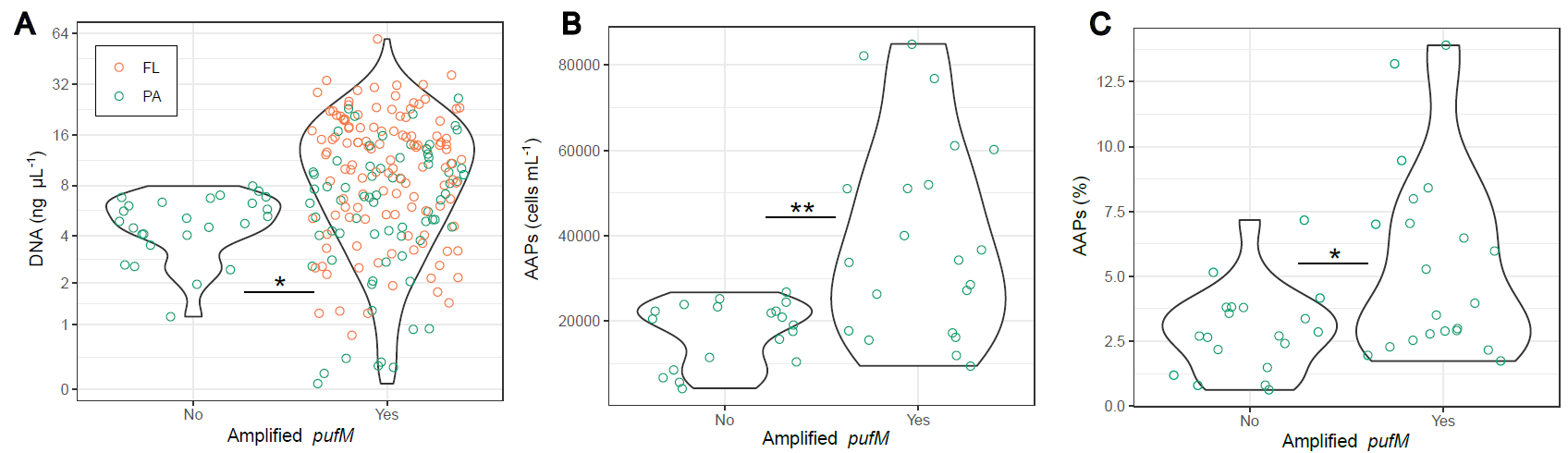


**Fig. S3.** (A) DNA yield (ng µL⁻¹, Qubit) by *pufM* amplification success, (B) AAP concentration (cell mL^-1^) by *pufM* gene amplification success, (C) AAP percentage of total bacteria (%) by *pufM* gene amplification success. Points are colored by fraction (FL = free-living, PA = particle-associated). Statistical significance: * *p* < 0.05, ** *p* < 0.01. Data correspond to values in Table S3.


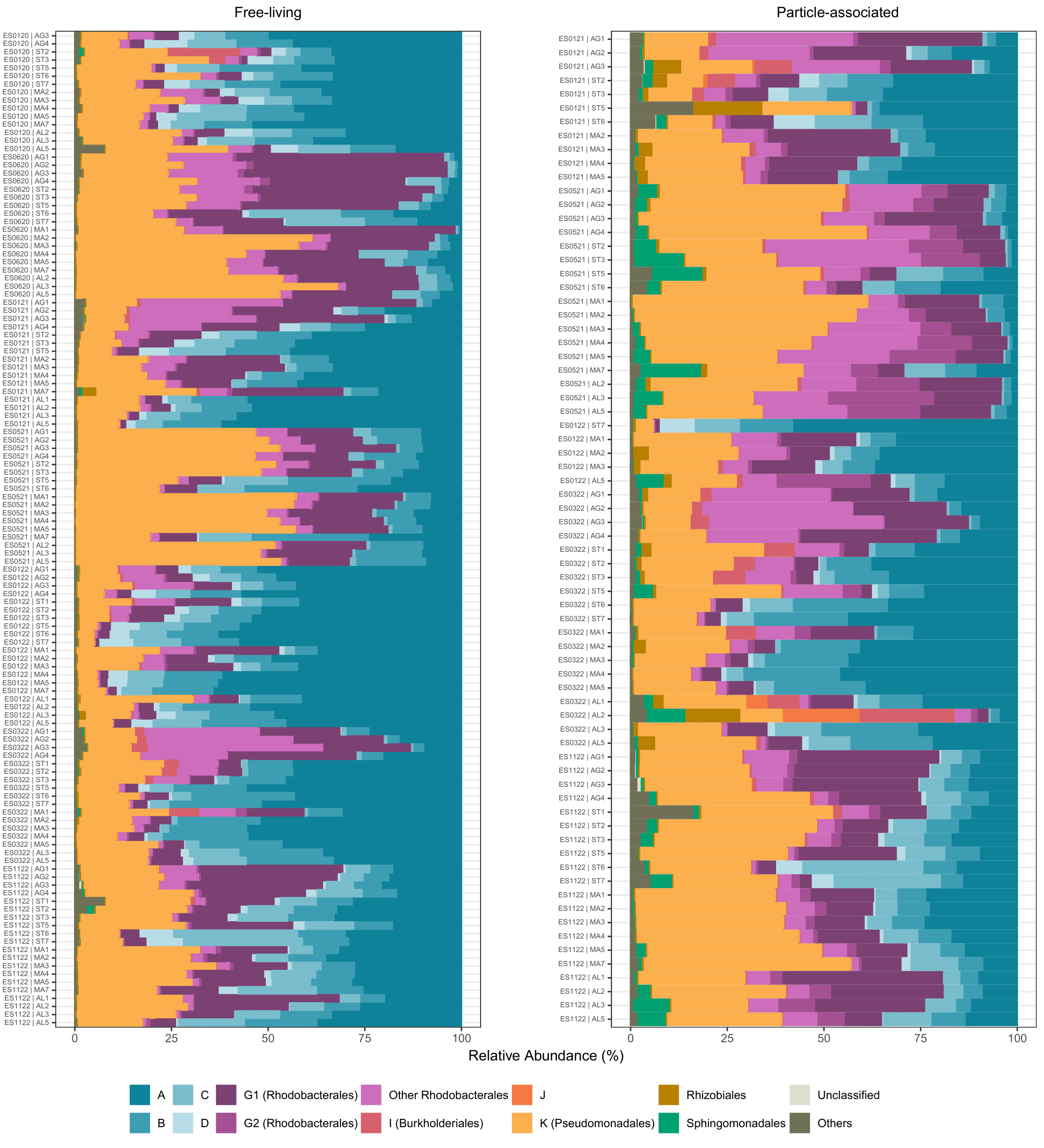


**Fig. S4.** Relative contribution of AAP phylogroups to total *pufM* gene reads at individual stations in the free-living (left panel) and particle-associated (right panel) size fractions. Samples are ordered chronologically by cruise, then arranged by transect from west to east, and then positioned along coast-to-offshore gradients.


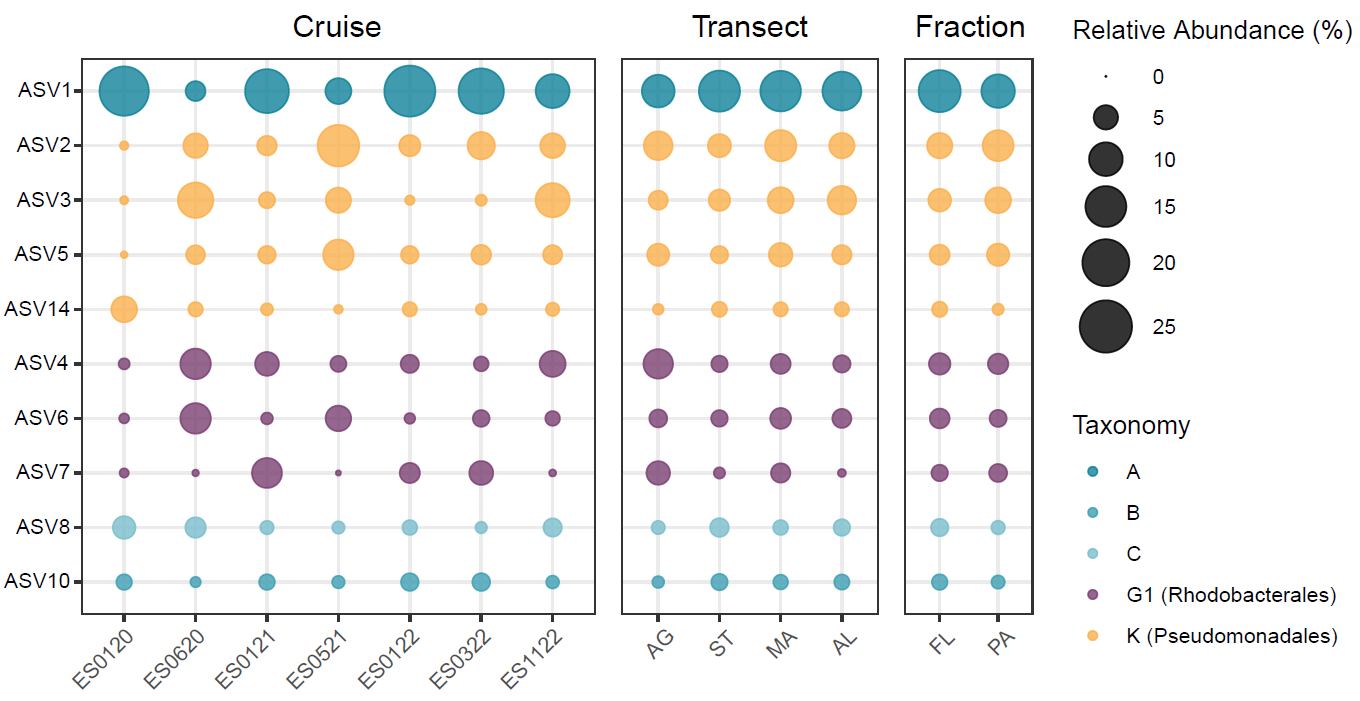


**Fig. S5.** Bubble plots showing the relative abundance of the top 10 ASVs across cruises (left), transects (middle), and size fractions (right). Bubble size represents the relative abundance of each ASV, and bubble color indicates their taxonomic affiliation.

**Supplementary Information:**

**SI1.** Aerobic Anoxygenic Phototrophic (AAP) bacterial abundance analyses using epifluorescence microscopy.

AAP bacterial abundances were determined following the protocol of Mašin et al., (2006) with modifications described in Piwosz et al., 2022. Cells were stained with 4’,6-diamidino-2-phenylindole (DAPI) and counted using an epiﬂuorescence Zeiss Axio Imager.D2 microscope equipped with a Plan-Apochromat 63/1.46 Oil Corr objective and a Collibri LED module illumination system (Carl Zeiss). All microscopy counts were performed at the Algatech Centre, Institute of Microbiology (CAS), Czech Academy of Sciences.

DAPI-stained total bacteria were visualized under blue excitation (325–370 nm) with emission at 420–470 nm. Chlorophyll *a* (Chl *a*) autofluorescence was recorded in the red part of the spectrum (450–490 nm excitation; 600–660 nm emission). Bacteriochlorophyll *a* (BChl *a*) and combined BChl *a* + Chl *a*‑containing cells were recorded in the infrared part of the spectrum (combined 325–370 nm, 450–490 nm, 545–565 nm and 615–635 nm excitation, 645–850 emission). To obtain net counts of BChl *a*–containing bacteria, the contribution of Chl *a*–containing organisms to the infrared image was subtracted following Cottrell et al. (2006). For each sample, five microphotographs were acquired and analyzed both manually and using ACMEtool2.0 (Bennke et al. 2016).

**References:**

Mašín M, Zdun A, Ston-Egiert J, Nausch M, Labrenz M, Moulisová V et al. Seasonal changes and diversity of aerobic anoxygenic phototrophs in the Baltic Sea. *Aquatic Microbial Ecology* 2006;**45**:247–54. <https://doi.org/10.3354/ame045247>

Piwosz K, Villena-Alemany C, Mujakić I. (2022). Photoheterotrophy by aerobic anoxygenic bacteria modulates carbon fluxes in a freshwater lake. *The ISME Journal* 2022;**16**:1046-1054. <https://doi.org/10.1038/s41396-021-01142-2>

Cottrell MT, Mannino A, Kirchman DL. Aerobic anoxygenic phototrophic bacteria in the Mid-Atlantic Bight and the North Pacific Gyre. *Applied and Environmental Microbiology* 2006;**72**:557–64. <https://doi.org/10.1128/AEM.72.1.557-564.2006>

Bennke CM, Reintjes G, Schattenhofer M, Ellrott A, Wulf J, Zeder M et al. Modification of a high-throughput automatic microbial cell enumeration system for shipboard analyses. *Applied and Environmental Microbiology* 2016;**82**:3289-3296. <https://doi.org/10.1128/AEM.03931-15>
